# The Conifer Root and Stem Rot Pathogen (*Heterobasidion parviporum*): Effectome Analysis and Roles in Interspecific Fungal Interactions

**DOI:** 10.3390/microorganisms7120658

**Published:** 2019-12-05

**Authors:** Zilan Wen, Zhen Zeng, Fei Ren, Fred O. Asiegbu

**Affiliations:** 1 Faculty of Agriculture and Forestry, P. O. Box 27, Latokartanonkaari 7, University of Helsinki, 00014 Helsinki, Finland; zilan.wen@helsinki.fi (Z.W.); zhen.zeng@helsinki.fi (Z.Z.); feifei3080@163.com (F.R.); 2 Experimental Center of Forestry in North China, Chinese Academy of Forestry, No. 1 Shuiza Road, Beijing 102300, China

**Keywords:** *Heterobasidion parviporum*, small secreted protein, interspecific fungal interaction, gene expression

## Abstract

*Heterobasidion parviporum* Niemelä & Korhonen is an economically important basidiomycete, causing root and stem rot disease of Norway spruce (*Picea abies* (L.) Karst) in Northern Europe. The *H. parviporum* genome encodes numerous small secreted proteins, which might be of importance for interacting with mycorrhiza symbionts, endophytes, and other saprotrophs. We hypothesized that small secreted proteins from *H. parviporum* (HpSSPs) are involved in interspecific fungal interaction. To identify HpSSP-coding genes potentially involved, we screened the *H. parviporum* effectome and compared their transcriptomic profiles during fungal development and in planta tree infection. We further conducted phylogenetic analysis, and identified a subset of hypothetical proteins with nonpredicted domain or unknown function as HpSSPs candidates for further characterization. The HpSSPs candidates were selected based on high-quality sequence, cysteine residue frequency, protein size, and in planta expression. We subsequently explored their roles during in vitro interaction in paired cultures of *H. parviporum* with ectomycorrhizal *Cortinarius gentilis*, endophytic *Phialocephala sphaeroides*, saprotrophs (*Mycena* sp., *Phlebiopsis gigantea*, and *Phanerochaete chrysosporium*), respectively. The transcriptomic profile revealed that a large proportion of effector candidates was either barely expressed or highly expressed under all growth conditions. In vitro dual-culture test showed that *P. sphaeroides* and *C. gentilis* were overgrown by *H. parviporum*. The barrage zone formation or no physical contact observed in paired cultures with the saprotrophs suggest they had either combative interaction or antibiosis effect with *H. parviporum*. Several HpSSPs individuals were up- or downregulated during the nonself interactions. The results of HpSSPs gene expression patterns provide additional insights into the diverse roles of SSPs in tree infection and interspecific fungal interactions.

## 1. Introduction

The basidiomycete *Heterobasidion parviporum* Niemelä & Korhonen is one of the most severe root rot pathogens colonizing Norway spruce (*Picea abies* (L.) Karst). *H. parviporum* root rot is common in southern Finland with annual economic losses estimated at 40 million euros for the Finnish forest industry [1,2]. Fresh stumps of spruce trees and wounds are the infection sites for aerial basidiospores released from perennial basidiocarps. The resident spores grow to vegetative mycelium, invading healthy neighboring trees via root contact [3]. Typically, the pathogen thrives as a necrotroph by killing living cells of its host trees, or as a saprotroph in deadwood cells. The woody resources also provide nutrients for endophytic and other saprotrophic fungi. For example, *Phlebiopsis gigantea* (Fr.) Jül shares the same ecological niche with *H. annosum* sensu lato (s.l.) by rapidly colonizing the stump surface for space and nutrients, which makes it an ideal biocontrol agent against *Heterobasidion* species [3,4]. Apart from *P. gigantea*, other saprotrophic fungi, mycorrhiza, and endophytes are also important sources of potential biocontrol agents. Many previous studies have focused on searching for new biocontrol agents, but very few investigated the role of small secreted proteins in influencing the outcome of the interaction.

Fungal competition in wood is commonly considered as either primary resource capture or secondary resource capture [5]. Primary colonizing species capturing territory in uncolonized resources generally possess characteristics of rapid germination and mycelial extension [5]. Secondary or later colonizers invading the colonized resources depend on lignocellulose utilization, tolerance to stress, and combative ability [5,6]. Antagonistic interaction between different fungal species either lead to entire replacement of one mycelium by another, or partial replacement, or deadlock, where neither mycelium can gain territory from the others [6,7]. The antagonistic interaction is always accompanied by production of extracellular enzymes, changes in gross morphology, and secretion of antagonistic metabolites such as volatile and diffusible organic compounds [5,7,8,9,10]. Laccases were induced during the interaction between *Trametes versicolor* and *Trichoderma harzianum* [8]. Nonself mycelial interaction caused changes in the profile of VOCs relative to self-pairings, both qualitatively and quantitatively [11]. Such morphological and metabolic changes are closely linked with modulation of gene expression. Microarray analysis of *T. versicolor* during interspecific mycelial interaction showed expression changes in genes related to cell wall biosynthesis, cell division, carbohydrate and nitrogen metabolism, glycine-rich RNA binding protein, playing wide roles in hyphal growth and stress response [12]. However, expression changes in genes coding for effectors or related to virulence from fungal pathogens remain to be explored during interspecific fungal interaction.

The availability of genome sequences of fungal pathogens has enabled the analysis of the expression level of numerous genes under diverse experimental conditions. The recent genome analysis of *H. parviporum* revealed that around 7% of genes were predicted to be secreted proteins (759 out of 10,502), known as the secretome [13]. A small proportion of the secretome of necrotrophic pathogens is believed to be necrotrophic effectors, which may be either toxic secondary metabolites or proteins that cause plant cell death during necrotrophic growth [14]. Effector prediction was performed principally on the basis that they were secreted from the fungal cell and expressed in planta [15], with relatively broad criteria, such as low molecular weight (MW) and rich in cysteine residues [16,17]. However, not all fungal effectors have low MW or are cysteine-rich [18]. In this study, we screened effector candidates from the secretome of *H. parviporum*, and selected high-priority candidates, named *H. parviporum* small secreted proteins (HpSSPs), from the predicted effectome based on protein size and the frequency of cysteine residues, or the upregulation profiles in planta. We hypothesized that HpSSPs would be involved in interspecific fungal interaction. The aims of the present study were: (1) characterization of transcriptomic profiles of HpSSPs during fungal development (conidiospores and free-living mycelia growth) and tree infection (saprotrophic and necrotrophic growth); (2) the modulation of a set of HpSSPs expression over the course of fungal interactions between *H. parviporum* and endophytic, mycorrhizal and other saprotrophic fungi in artificial media.

## 2. Materials and Methods

### 2.1. Fungal Sampling and RNA Isolation in Paired Cultures

All fungal isolates (*Heterobasidion parviporum* 96026 HAMBI 2359, *Phialocephala sphaeroides* strain 222, *Stereum sanguinolentum* FBCC 1148, *Phlebiopsis gigantea* (courtesy of Kari Korhonen), *Phanerochaete chrysosporium* HE-446 FBCC 280, *Mycena* sp. JH226, *Cortinarius gentilis* K94 FBCC 546) were maintained on malt extract agar (MEA) (malt extract 20 g/L, agar 15 g/L). Homokaryotic *H. parviporum* strain 96026 (Åland, Finland) was provided by courtesy of Kari Korhonen. *P. sphaeroides* in this study, originally isolated from roots of Norway spruce from minerotrophic pristine mire (*Vaccinium myrtillus* spruce swamp (MK) [19], is a dark septate root endophyte. All other isolates were obtained from the University of Helsinki Fungal Biotechnology Culture Collection (HAMBI/FBCC). We grouped the six fungal species into the endophytic fungus (*P. sphaeroides*), saprotrophic fungi (*S. sanguinolentum*, *P. gigantea*, *P. chrysosporium*, *Mycena* sp.), the ectomycorrhizal fungus (*C. gentilis*) according to their lifestyles. Each fungal species was cocultured with *H. parviporum* on MEA in a 90 mm diameter petri dish. Two agar plugs (5 mm diameter) were placed at a distance of 60 mm on MEA surface preoverlaid with a piece of cellophane membrane. *H. parviporum* self-pairing (HpHp) was used as an additional control. The fungi were grown at 20 °C in darkness. Mycelia from the paired cultures were collected at different interaction stage, including precontact (t1), initial contact (t2) (i.e., the first time when mycelia from two opposing fungi meet each other in paired cultures), and days postcontact (t3) (i.e., the situation where overgrowth or barrage zone occurred several days post initial contact). There were three biological replicates at each interaction stage, with each replicate consisting of mycelia from different MEA plates. Total RNA was isolated by using TRI reagent (Sigma Aldrich), following the manufacturer’s instructions [20].

### 2.2. Effectome Analysis

#### 2.2.1. Effectome Screening, Phylogenetic Analysis of Effectome, and HpSSP-Coding Genes Selection

The sequenced genome of *H. parviporum* 96026 was used to identify the effectome. The genome contains approximately 10,500 predicted genes, 287 scaffolds, and 759 secreted proteins [13]. Subsequently, the predicted secretome was submitted to EffectorP v.1.0 [15] and LOCALIZER v.1.0. EffectorP was used to prioritize the selection of highly confident effector candidates. LOCALZIER aids the prediction of translocation of fungal effectors into plant chloroplasts, mitochondria, and nuclei based on the presence of transit peptides and nuclear localization signals. In addition, secreted proteins with length less than 150 amino acids and number of cysteine residues greater than three were also selected. All three sets of protein lists were merged and constituted the final effectome after removing the putative CAZymes, peroxidases, and proteases. The resulting protein sequences were aligned by ClustalW in MEGA v.7. Neighbor-Joining (NJ) was performed in MEGA v.7 for phylogenetic analysis in the predicted effectome.

We filtered out a small number of candidates with incorrect gene structures (i.e., genes not supported by transcript data). Twelve small secreted proteins (HpSSPs) were selected from the remaining effectome based on the length of amino acids, the percentage of cysteine residues, or expression patterns at diverse developmental stages.

#### 2.2.2. Effectome Expression Pattern at Diverse Developmental Stages

Expression of *H. parviporum* effector candidates was monitored during developmental stages of the fungus: conidiospores, free-living mycelia, saprotrophic, and necrotrophic growth. The methods of sample preparation and collection were as previously described [21]. Briefly, conidiospores were collected from mycelia pregrown on Hagem agar for three weeks. Free-living mycelia were obtained from conidiospores pregrown for three weeks in Hagem liquid medium. Samples for saprotrophic growth were obtained from sawdust precolonized by *H. parviporum* within the inoculated trees during in planta growth, whereas samples for necrotrophic growth were obtained from necrotic lesions surrounding living cells of the inoculated trees after three and half months [21].

### 2.3. cDNA Synthesis

Complementary DNA (cDNA) was synthesized according to the Thermo-Fisher Scientific’s protocol. We added 1 µL of DNase I (1 U/ µL, ThermoFisher Scientific, Waltham, MA, USA) to 1 µg of total RNA to remove genomic DNA and subsequently deactivated by heating at 65 °C for 10 min in the presence of 1 µL of 50 mM EDTA. A total of 100 µM Oligo(dT)_18_ Primer (1 µL, 100 µM, ThermoFisher Scientific, Waltham, MA, USA) was mixed with the DNA-free RNA template at 65 °C for 5 min for primer annealing and then incubated on ice. After annealing, the reverse transcriptase and components including buffer, dNTPs, and RNase inhibitor were added. The reaction mixture was incubated at 42 °C for 60 min and the reaction was stopped by heating at 70 °C for 5 min.

### 2.4. Quantitative Real-Time PCR

We designed the primers for qPCR at Roche Universal Probe Library Assay Design Center (https://lifescience.roche.com/en_fi/brands/universal-probe-library.html#assay-design-center). The cDNA template (3 µL) was diluted into 97 µL of nuclear-free water. The total qPCR reaction volume was 15 µL, including 5.5 µL of the diluted cDNA, 1 μL of forward primer (10 μm), 1 μL of reverse primer (10 μm), and 7.5 μL of two times LightCycler 480 SYBR Green I Master (Roche, Espoo, Finland). The reaction program was as follows: 5 min denaturation at 95 °C, 45 amplification cycles of 10 s at 95 °C, 10 s annealing at 60 °C, and extension 20 s at 72 °C. The relative quantities of HpSSP-coding genes were normalized with four housekeeper genes in *H. parviporum*, including RNA polymerase III transcription factor (RNA Pol3 TF), mannosyltransferase (Maan transf), tryptophan catabolism (Tryp metab), ribosomal protein S23 (RiboS23). All primers used in this study are shown in Table S1.

## 3. Results

### 3.1. Effectome Analysis

The predicted *H. parviporum* effectome initially contained 268 proteins, as seen in Table S2A). A total of 163 effector candidates, as seen in Table S2B, remained after excluding genes encoding cytochrome P450, hydrophobins, and candidates with wrong gene structures (i.e., not supported by RNA transcripts data). Approximately 40% of the proteins (67 out of 163) were hypothetical proteins. EffectorP recognized 40 candidates as predicted effectors, as seen in Table S2C. A recent study provided transcriptomic data of *H. parviporum* infecting mature Norway spruce, and showed that 1060 genes were expressed in planta [21]. In this study, we found that 17 out of the 163 effector candidates were on this gene list, as seen in Table S2D. Three predicted effectors (evm.scaffold 22.83, 34.58, and 6.140) displayed in planta expression.

**Figure 1:**
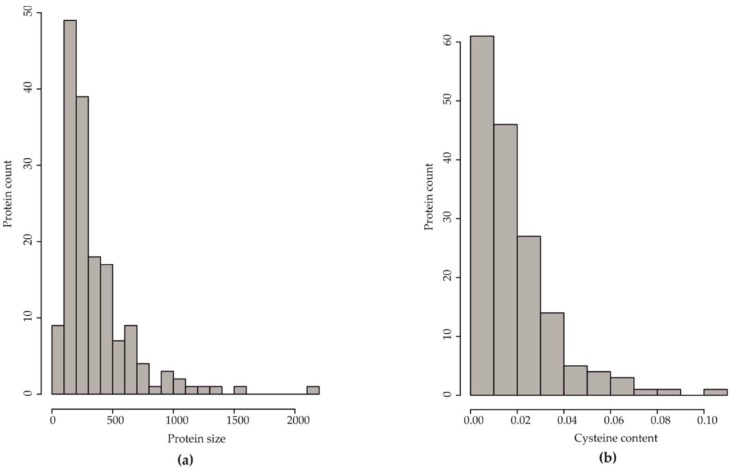
(**a**) Distribution of the length of predicted *H. parviporum* effector candidates (protein size estimated as the number of amino acids). (**b**) Distribution of cysteine frequency in predicted *H. parviporum* effector candidates. Cysteine frequency refers to the number of cysteine residues per hundred amino acids.

Among the 163 effector candidates, 97 candidates (59.5%) contained less than 300 aa, with the largest group containing 100–200 aa (49 candidates), followed by those between 200 aa and 300 aa (39 candidates). The number of proteins with the size between 300 to 400 aa was approximately equal to those of size 400–500 aa, 18 and 17 candidates, respectively. Only a small number of proteins (24 candidates) had the length of 500–1000 aa, making up 14.7%. Only seven candidates had over 1000 aa, as seen in Figure 1a. Apart from the protein size, the percentage of cysteine residues is one of the discriminative features in effector classification [15]. We found 61 out of 163 proteins had ≤1% cysteine (one cysteine residue per 100 aa), with 26 proteins without cysteine residues, as seen in Figure 1b, Table S2B. The proteins containing 1–2%, 2–3%, and 3–4% cysteine residues (46, 27, 14 out of 163, respectively), together with ≤1% cysteine residue proteins, comprised nearly 90% of the effectome.

Since a large proportion of the effector candidates consisted of hypothetical proteins with unknown function, phylogenetic analysis was utilized to explore the potential functional group of the hypothetical proteins based on their sequence similarity to proteins of known function. The phylogenetic analysis showed that 163 effector candidates were distinctly divided into four main groups, as seen in Figure S1. The twelve effector candidates were further chosen based on one of the following criteria: (1) upregulation during saprotrophic growth or necrotrophic growth; (2) less than 300 amino acids and an even number of cysteine residues; and (3) uncharacterized hypothetical proteins. We selected 12 hypothetic proteins among the four groups, regarded as small secreted proteins of *H. parviporum* (HpSSPs). Their protein size, the cysteine residue frequency, and the homologs *H. irregulare* are shown in Table 1. HpSSP33.48 shared high similarity with HpSSP33.91 in terms of protein size, the position and number of cysteine residues, a homolog in *H. irregulare*, as seen in Table 1, which resulted in the location of the two proteins at the same branch in the phylogenetic tree.

### 3.2. Transcriptomic Profiles of the Effector Candidates During Fungal Development and in Planta Expression

**Figure 2:**
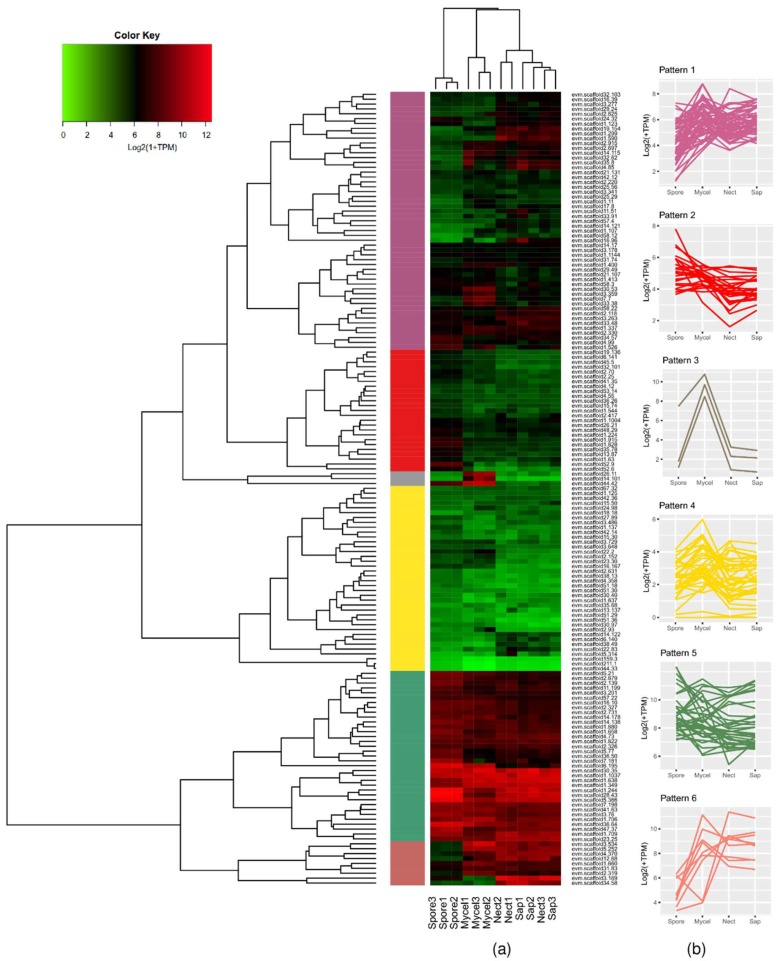
(**a**) Hierarchical clustering of effector candidates by Log2-transformation of (1 + TPM) under different growth conditions: *H. parviporum* as conidiospores (Spore), free-living mycelia (Mycel), saprotrophic sawdust growth (Sap), and necrotrophic growth in the stem tissue (Nect). (**b**) We divided expression of effector candidates into six patterns as shown in the heatmap.

With the sequenced RNAs from free-living mycelia (MYCEL), conidiospores (SPORE), saprotrophic sawdust growth (SAP), and necrotrophic growth in the necrotic stem tissue (NECT) in a previous study [21], we investigated the transcriptomic profiles of effector candidates. All effector candidates were divided into six groups based on the hierarchical clustering of their expression patterns, as seen in Figure 2. Genes barely expressed or highly expressed under all conditions were clearly separated into pattern 4 and pattern 5, respectively. A small subset of candidates, which were highly expressed under all conditions except SPORE or highly expressed in both NECT and SAP, was regarded as pattern 6. Candidates weakly expressed only in SPORE were in pattern 2. Only three candidates were expressed during fungal development (pattern 3). The rest of the candidates without preference for expression condition were grouped into pattern 1. However, in-planta expressed candidates, which were highly upregulated in both NECT and SAP and weakly expressed in SPORE and MYCEL, did not belong to any single group. Among the twelve selected HpSSPs, five individuals were in pattern 1 with irregular expression pattern. HpSSP26.11, in pattern 3, was expressed only in MYCEL. HpSSP2.152 and HpSSP3.729 were barely expressed under all conditions, belonging to pattern 4. HpSSP1.244 and HpSSP2.326 were expressed under all conditions, belonging to pattern 5. HpSSP3.534 was expressed under all conditions except SPORE, and HpSSP3.169 was expressed both in NECT and SAP. Both of them were grouped into pattern 6.

**Figure 3:**
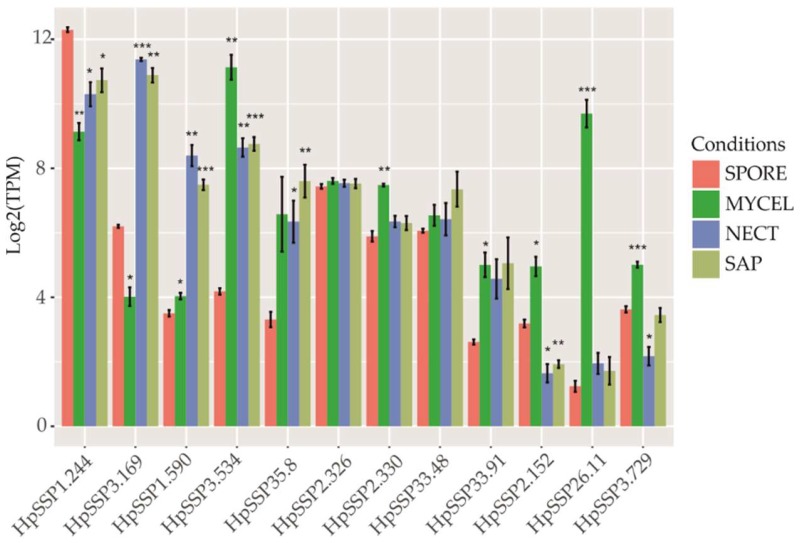
Transcriptome profile of HpSSP-coding genes from free-living mycelia (MYCEL), saprotrophic sawdust growth (SAP), necrotrophic growth in the necrotic wood tissue (NECT), and conidiospores (SPORE). Error bars represent the SD from three independent experiments. Asterisks indicate significant difference from the level under SPORE condition (*** *p* < 0.001; ** *p* < 0.01; * *p* < 0.05).

We closely inspected whether the selected HpSSP-coding genes were significantly expressed in NECT and SAP compared to SPORE. HpSSP1.244, as a predicted effector, as seen in Table 1, showed a considerably high expression level under all conditions, with a significant difference compared to the level in SPORE, as seen in Figure 3. HpSSP3.169 and HpSSP1.590, as predicted noneffectors, as seen in Table 1, displayed surprisingly significant induction in both NECT and SAP. HpSSP3.534 was dramatically expressed in MYCEL, NECT, and SAP, compared to SPORE. HpSSP35.8, predicted as an unlikely effector in silico, was significantly induced in NECT and SAP. The expression level of HpSSP2.326, HpSSP2.330, HpSSP33.48, and HpSSP33.91 showed no significant difference in NECT and SAP from those in SPORE. HpSSP2.152, HpSSP26.11, and HpSSP3.729 were barely expressed in NECT and SAP, with no significant difference compared to SPORE, while they were significantly induced in MYCEL.

### 3.3. Mycelial Interaction in Dual Cultures

**Figure 4:**
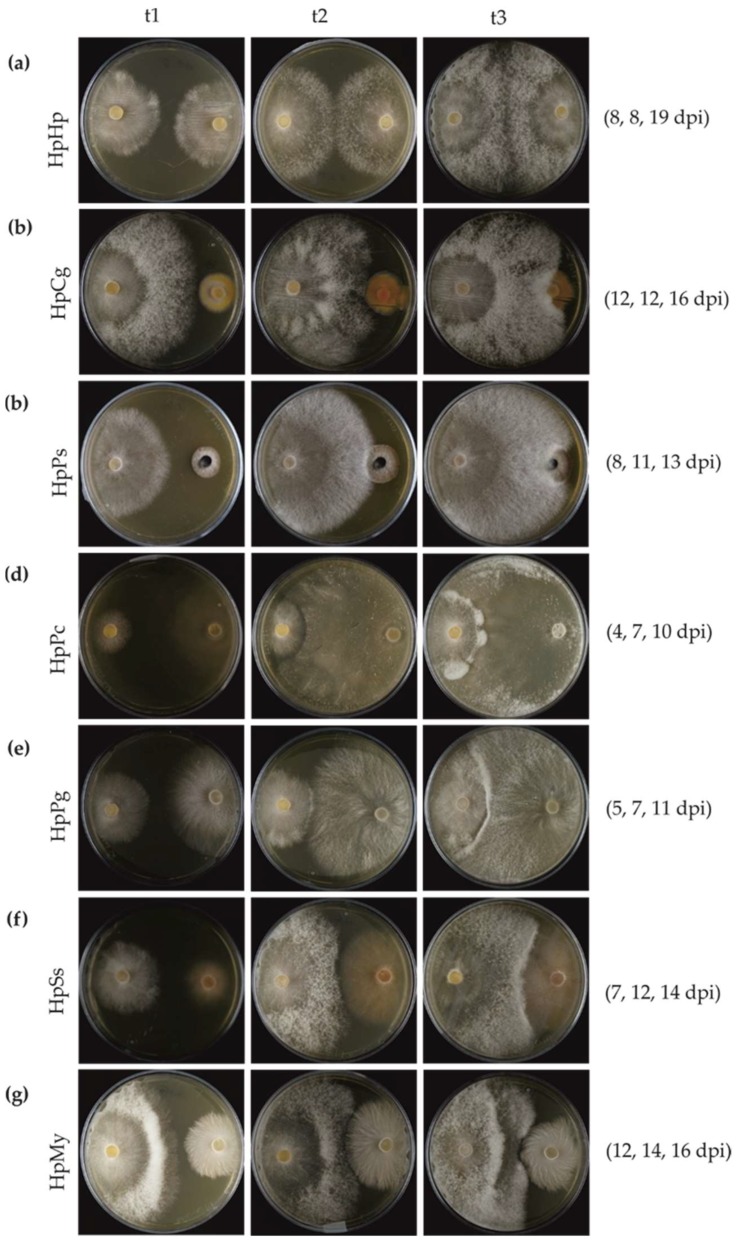
Mycelial interaction between *H. parviporum* (left side of Petri plates) and other fungi (right side of the plates) in paired cultures. Mycelia from the paired cultures were collected at precontact (t1), initial contact (t2), and postcontact (t3). (**a**) Self-interaction between *H. parviporum* (HpHp), both pre- and initial contact (t1 and t2) with *H. parviporum* occurred at 8 days postinoculation (dpi), t3 = 19 dpi. (**b**) Overgrowth during interaction between *H. parviporum* and *C. gentilis* (HpCg). Mycelia collection at t1, t2 (12 dpi), and t3 (16 dpi). (**c**) Overgrowth during interaction between *H. parviporum* and *P. sphaeroides* (HpPs). t1: 8 dpi; t2: 11 dpi; t3: 13 dpi. (**d**) Partly replacement of *H. parviporum* for *P. chrysosporium* (HpPc). t1: 4 dpi; t2: 7 dpi; t3: 10 dpi. (**e**) Barrage zone formed during interaction with *P. gigantea* (HpPg). t1: 5 dpi; t2: 7 dpi; t3: 11 dpi. (**f**) Barrage zone formed during interaction with *S. sanguinolentum* (HpSs). t1: 7 dpi; t2: 12 dpi; t3: 14 dpi. (**g**) Distance during interaction with *Mycena* sp. (HpMy). t1: 12 dpi; t2: 14 dpi; t3: 16 dpi.

The interaction outcome was not only dependent on the species combinations, but also partly on the growth rate of the fungal isolates. This led to differences in the time points at precontact (t1), initial contact (t2), and postcontact (t3). *P. chrysosporium* and *P. gigantea* grew much faster, such that the initial contact with *H. parviporum* was earlier than for the other tested fungi. *H. parviporum* grew much faster than *C. gentilis* and *P. sphaeroides*, accumulating much more biomass. The time of precontact (t1) and initial contact (t2) in self-interaction was at 8 days postinoculation (dpi). The mycelia of *H. parviporum* self-pairing (HpHp) postcontact (t3) was collected at 19 dpi, as seen in Figure 4a. During the interaction between *H. parviporum* and *C. gentilis* (HpCg), mycelia at t1 and t2 were harvested at 12 dpi, and the mycelia at t3 were collected at 16 dpi, as seen in Figure 4b.

The self-pairing produced uniform mycelial mats, and no barrage zone was formed, as seen in Figure 4a. Nonself interactions could be generally classified either into overgrowth, barrage zone, or antibiosis at a distance. Both C. *gentilis* and *P. sphaeroides,* which developed at much slower speed than *H. parviporum,* were easily overgrown by the conifer pathogen, as seen in Figure 4b,c. Dense mycelia were formed at the interaction zone in *H. parviporum* vs. *P. chrysosporium* (HpPc), *H. parviporum* vs. *P. gigantea* (HpPg), and *H. parviporum* vs. *S. sanguinolentum* (HpSs), as seen in Figure 4d–f. *H. parviporum* produced mycelia that partly invaded into the territory occupied by *P. chrysosporium*, as seen in Figure 4d. A barrage zone was formed in HpSs. However, unlike the situation mentioned above, no hyphal contact occurred in the interaction between *H. parviporum* and *Mycena* sp. (HpMy). Antagonism or antibiosis between fungi without hyphal contact can be mediated via volatile or diffusible chemicals or other antifungal metabolites [5]. Barrage zone formation or no hyphal contact implied that *P. chrysosporium*, *P. gigantea*, *S. sanguinolentum*, and *Mycena* sp. might have a competitive impact on the growth of *H. parviporum*.

### 3.4. Gene Expression of HpSSPs on Dual Cultures

**Figure 5:**
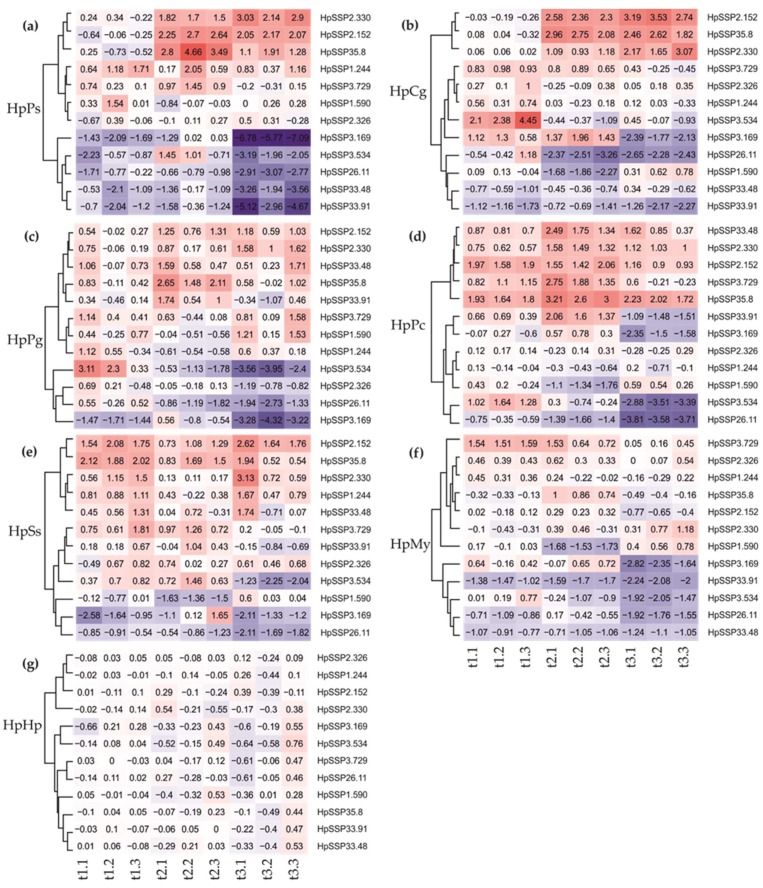
Expression analysis of HpSSP-coding genes in *H. parviporum* during interspecific interactions. Normalized relative quantification (NRQ) of HpSSP-coding genes in nonself interactions relative to self-interaction between *H. parviporum* 96026 isolates (HpHp). Four reference genes were used: RNA Pol3 TF, Maan transf, Tryp metab, and RiboS23. Mycelia from the paired cultures were collected at precontact (t1), initial contact (t2), and postcontact (t3). HpCg: *H. parviporum* vs. *C. gentilis.* HpMy: *H. parviporum* vs. *Mycena* sp. HpPc: *H. parviporum* vs. *P. chrysosporium*. HpPg: *H. parviporum* vs. *P. gigantea.* HpPs: *H. parviporum* vs. *P. sphaeroides.* HpSs: *H. parviporum* vs. *S. sanguinolentum.*

To investigate the gene expression pattern of the selected HpSSPs responsive to the tested endophyte, ectomycorrhiza, and saprotrophic basidiomycetes, the normalized relative quantities of the 12 selected HpSSP-coding genes were assessed at the precontact (t1), initial contact (t2), and postcontact (t3) during the interspecific fungal interactions. When the HpSSPs expression profiles of each pairwise combination were clustered, HpSSP2.330, HpSSP2.152, and HpSSP35.8 were upregulated during the nonself interactions, except in HpMy, while HpSSP26.11, HpSSP3.169, and HpSSP3.534 were commonly downregulated, especially at t3, as seen in Figure 5. However, their expression level depended on the hyphal interaction stages and the interacting species. Notably, HpSSP expression in HpMy was overall less active compared to other combinations, as seen in Figure 5f.

During the interaction between *H. parviporum* and the endophyte *P. sphaeroides* (HpPs), we found strong regulation of the commonly expressed HpSSP individuals, compared to the combination with other wood-decay basidiomycetes, as seen in Figure 5a. Additionally, HpSSP33.48 and HpSSP33.91 were also downregulated in HpPs particularly at t3, as seen in Figure 5a, which was different from other interactions. Ectomycorrhizal *C. gentilis* was also replaced by the pathogen on malt extract agar, and HpCg likely shared the similar expression dynamics to HpPs, including the commonly upregulated ones (HpSSP2.330, HpSSP2.152, and HpSSP35.8), HpSSP26.11, HpSSP33.48, and HpSSP33.91, as seen in Figure 5b. HpSSP3.534 was induced at t1, while HpSSP3.169 was induced at t1 and t2, and downregulated at t3. HpSSP1.590 was inactive in HpPs, but downregulated at t2 in HpCg, as seen in Figure 5b.

Interactions with white-rot *H. parviporum* were also investigated in pairwise combinations with three wood-decaying basidiomycetes. Dense mycelia were formed at the interaction zone in *H. parviporum* vs. *P. chrysosporium* (HpPc), as seen in Figure 4d, *H. parviporum* vs. *P. gigantea* (HpPg), as seen in Figure 4e, and *H. parviporum* vs. *S. sanguinolentum* (HpSs), as seen in Figure 4f. Among those fungi, we expected that white-rot fungal species *P. chrysosporium* and *P. gigantea* should be more aggressive against *H. parviporum* based on the less biomass produced by *H. parviporum*. Unlike in HpPs and HpCg, the expression of HpSSP33.48 in HpPg and HpPc was upregulated over the observed period, as seen in Figure 5c,d. HpSSP33.91 was weakly expressed at t2 in HpPg, and it was also upregulated at t2 but downregulated at t3 in HpPc. Both of them were relatively inactive in HpSs, as seen in Figure 5e. This result might reflect that HpSSP33.48 and HpSSP33.91, despite the similarity in protein sequence, might be functionally different. HpSSP35.8 was expressed only at t2 in HpPg, as seen in Figure 5c, while it was expressed at all time points in HpPc, as seen in Figure 5d, and HpSs, as seen in Figure 5e. HpSSP1.244, which was downregulated at t2 in HpPg, as seen in Figure 5c, and HpPc, as seen in Figure 5d, was weakly upregulated at t1 and t3 in HpSs, as seen in Figure 5e. HpSSP3.729, relatively inactive in HpPg, was strongly induced at t2 in HpPc and slightly expressed at t1 and t2 in HpSs. Unlike other combinations of mycelial contact, the interaction between *H. parviporum* and saprotrophic *Mycena* sp. (HpMy) was antagonism at a distance, as seen in Figure 4g. HpSSPs which were commonly upregulated in other combinations appeared to be inactive in HpMy. In HpMy, HpSSP3.729 was weakly expressed at t1 and t2, and HpSSP35.8 was slightly upregulated at t2. The commonly downregulated HpSSPs, along with HpSSP33.91 and HpSSP33.48, were silent especially at t3 in HpMy, as seen in Figure 5f.

## 4. Discussion

Interspecific fungal interaction, as a key feature of fungal community formation, dramatically affects the size of the populations and the fitness of individuals. Fungal competition is commonly applied in screening for potential biocontrol agents against phytopathogens of trees. *H. parviporum*, as a necrotrophic fungal pathogen of Norway spruce, has intimate interactions with other fungal species, such as endophytes and mycorrhiza during invasive growth. In our pairwise combinations, hyphal intermingling was observed in self-pairings, which may result from cooperative or neutral interaction [22]. *H. parviporum* overgrew endophytic *P. sphaeroides* and mycorrhizal *C. gentilis* in this study. However, the root endophyte *P. sphaeroides* was considered to possess inhibitory capability to reduce the growth of *H. parviporum* [23]. The dominance of *H. parviporum* overwhelmed the mycelial growth of *P. sphaeroides*, which might have affected its inhibitory effect. A barrage zone was formed when *H. parviporum* was paired with *P. chrysosporium*, *P. gigantea* [24], and *S. sanguinolentum*. The barrage zone formation in HpPg was temporary, as overgrowth of *H. parviporum* by *P. gigantea* was observed during prolonged incubation. Mycelial growth of *H. parviporum* encountered an antibiosis effect when paired with *Mycena* sp., possibly due in part to the effects of diffusible chemical production [25]. Barrage zone formation or no physical contact suggest that combative or antagonistic interactions occurred. The availability of *H. parviporum* genome sequences has provided a much better understanding of the regulation of small secreted proteins (HpSSP) possibly involved in pathogenicity, as well as in the fungal interaction under those pairwise combinations. We found that a subset of HpSSPs shared similar expression dynamics over interaction combinations, while some HpSSPs showed combination-dependent expression. Although the identity of the HpSSPs documented in our study is still unknown, this does not diminish their functional relevance.

Diverse strategies utilized by fungal individuals might confer flexibility towards local changes in the environment to increase their fitness, which may be partly derived from elaborately regulated gene expression profiles in response to diverse conditions [26]. Fungi have hundreds or even over a thousand predicted secreted proteins, among which the proportion of SSPs in most fungi is 25% to 60% across all lifestyles [27,28,29]. In this study, *H. parviporum* was predicted to have 21.4% HpSSPs in its secretome (163/759). This percentage is greater than the proportions of effectors in the secretomes of other white rot fungi, such as *T. versicolor* (13.1%), *Dichomitus squalens* (11.2%), and *Stereum hirsutum* (8.5%) [15]. Here, we analyzed the transcriptomic profiles of *H. parviporum* effector candidates during fungal growth in wood sawdust (SAP), fungal invasion in necrotic stem tissues (NECT) and the profile in conidiospores (SPORE), and mycelial growth on artificial media (MYCEL). The results showed a large proportion of effector candidates were either barely expressed or highly expressed under all growth conditions. A few candidates were upregulated in both NECT and SAP relative to SPORE and MYCEL, including HpSSP3.169 and HpSSP1.590. No NECT-specific gene was observed in the transcriptome analysis. Effector proteins are believed to be temporally expressed, with a set of effectors highly expressed during the early infection and some highly expressed at the later stages [30,31]. In our previous study [21], necrotic tissue was collected after three and a half months of infection, and some effectors were probably already expressed before the sampling. Therefore, the low expression in SAP and NECT observed in the study does not imply that they were not expressed during the entire infection stages. Moreover, we found similar expression patterns in SAP and NECT, which might be due, in part, to the fact that the fungus in necrotic stem tissue might have already switched from necrotrophic to saprotrophic growth.

Although HpSSP3.169 and HpSSP1.590 were predicted noneffectors, they were highly expressed in SAP and NECT. Their roles in pathogenicity during host–pathogen interaction need to be further explored. HpSSP1.244 showed considerably high induction in SPORE, MYCEL, SAP, and NECT, which indicated that it might contribute to all lifestyles of the pathogen. HaSSP1, a homolog of HpSSP1.244 in *H. annosum*, also had a high transcript level in both saprotrophic and necrotrophic conditions, as well as in liquid media [32]. HpSSP3.534 and HpSSP35.8 were upregulated in free-living mycelia and in planta, suggesting that they might be potentially involved in fungal development and pathogenicity. HaSSP16, homolog of HpSSP3.534 in *H. annosum*, showed low transcript level in necrotrophic conditions, but high transcript level in saprotrophic growth in wood [32]. HpSSP35.8 probably had a role in the disease process based on its high expression in the presymptomatic phase of seedling infection, and it induced SSP-associated programmed cell death in *Nicotiana benthamiana* [33]. The expression level of HpSSP33.48 was higher than that of HpSSP33.91, especially in SPORE, which revealed that they are probably different in function. Compared to SPORE, HpSSP2.152, HpSSP26.11, and HpSSP3.729 were significantly induced in MYCEL and weakly expressed in SAP and NECT. The expression situation might be associated with the presence of glucose or other easily metabolizable sugars in the cultivation media. Wood-degrading basidiomycete fungi fine-tune enzyme production in response to carbon sources [26,34]. The regulation pattern of genes related to carbohydrate-active enzymes produced by the brown rot fungus *Postia placenta* grown on cellulose was distinct compared to growth on glucose [35]. We reasoned that the variation of HpSSPs expression in MYCEL and SAP and NECT was likely due to carbon availability, with glucose and maltose in Hagem media and high cellulose and lignin content in wood substrates.

The regulation of small secreted proteins (HpSSP) from the forest pathogen *H. parviporum* during interspecific interaction has been poorly documented thus far [24,36,37]. Here, we analyzed the regulation of the fungal HpSSP-coding genes at several stages of interaction (precontact, initial contact, postcontact) with endophytes, mycorrhiza, and other saprotrophs. Gene expression of individual HpSSPs were dramatically altered in response to interspecific fungal interactions. The tree pathogen *H. parviporum* responded with both common and combination-dependent HpSSP regulation when interacting with different fungal species. A mycoparasitic fungus, *Clonostachys rosea*, responded with both common and specific gene expression during interaction with the plant pathogen *Botrytis cinerea* and *Fusarium graminearum*. The common responses in *C. rosea* included sugar and small organic compound transporters potentially involved in nutrient uptake. The specific responses against the fungal pathogens were dominated by genes associated with membrane transport, biosynthesis of secondary metabolites, and carbohydrate degradation [38]. During mycorrhiza–helper bacteria interaction, the early upregulation of bacteria-responsive genes could be linked to bacterial recognition, growth, and morphological modifications, such as towards fungal lipid anabolism and catabolism, while in the late stage, the responsive genes encode proteins of primary metabolism [39]. After extended contact, genes involved in protein synthesis and degradation were repressed. The repression of the protein synthesis machinery may reveal a reduction in the transcription of several genes involved in energy metabolism [39]. In the current study, interpreting the roles of the regulation of HpSSP-coding genes in each fungal interaction is difficult, due in part to the fact that they are hypothetical proteins with unpredicted domains or unknown function. Some of HpSSP-coding genes upregulated in the early stage of interaction (at the precontact and the initial contact) might function in fungal recognition and nutrient uptake. Some HpSSP-coding genes downregulated postcontact might suggest a reduction of energy metabolism. The interaction between *H. parviporum* and *P. gigantea* has been widely studied, since *P. gigantea* is commonly used as a biocontrol agent because of its competitive advantage against *H. parviporum* [40]. Hyphal interference of cells of *H. annosum* in contact with the hyphae of *P. gigantea* has been documented in dual cultures in agar medium [41]. A number of *H. annosum* genes involved in glucose metabolism transport, signal transduction, and defense were differentially downregulated after exposure to *P. gigantea* culture filtrate. This might indicate that the nutrient processing, acquisition, and resistance machinery of *H. annosum* were suppressed by *P. gigantea* during antagonistic interaction [36]. Our results have highlighted the varying roles of a subset of small secreted proteins of the conifer pathogen during interspecific interaction with a diverse number of ecologically important forest fungi. Further insight on the regulatory patterns of the genes encoding the effector-like proteins during the interspecific interaction will help in our understanding of this complex ecological process.

## Figures and Tables

**Table 1:** Summary of the selected *H. parviporum* small secreted proteins (HpSSPs).

HpSSP	Homolog to *H. annosum*.s.s ^a^	Sequence Length	Cysteins Number	Prediction in EffetorP	Transcriptomic Pattern
HpSSP1.244	Hetan1.estExt_Genewise1Plus.C_10476	194	10	Effector	Pattern 5
HpSSP2.152	Hetan1.Genemark.3574_g	152	4	Effector	Pattern 4
HpSSP2.326	Hetan1.estExt_Genewise1Plus.C_40816	242	12	Effector	Pattern 5
HpSSP2.330	fgenesh1_pm.03_#_457	192	2	Effector	Pattern 1
HpSSP26.11	e_gw1.11.833.1	254	12	Noneffector	Pattern 3
HpSSP3.534	Hetan1.Genemark.10137_g	185	10	Effector	Pattern 6
HpSSP3.729	Hetan1.gw1.14.303.1	216	4	Effector	Pattern 4
HpSSP33.48	Hetan1.fgenesh2_pg.C_scaffold_13000272	243	4	Effector	Pattern 1
HpSSP33.91	Hetan1.fgenesh2_pg.C_scaffold_13000272	245	4	Effector	Pattern 1
HpSSP35.8	estExt_fgenesh1_pg.C_130285	177	2	Unlikely effector	Pattern 1
HpSSP1.590	Genemark.573_g	321	0	Noneffector	Pattern 1
HpSSP3.169	gw1.07.1673.1	524	0	Noneffector	Pattern 6

^a^https://mycocosm.jgi.doe.gov/Hetan2/Hetan2.home.html.

## Supplementary Materials

The following are available online at https://www.mdpi.com/2076-2607/7/12/658/s1. Figure S1: Phylogenetic relationships of predicted effector candidates among *H. parviporum*. A total of 163 protein sequences were aligned by ClustalW and were used to for phylogenetic analysis by using the neighbor-joining method in the MEGA v.7. A total of 12 protein sequences for gene expression study during interspecific fungal interaction were highlighted in blue, Table S1: Primers for HpSSP-coding genes in this study [42], Table S2: Summary of effectome candidates.
