# Supplementary figures and images for: The Conifer Root and Stem Rot Pathogen (Heterobasidion parviporum): Effectome Analysis and Roles in Interspecific Fungal Interactions
Source: Microorganisms. 2019 Dec 5;7(12):658. doi: 10.3390/microorganisms7120658 (PMC6955712; doi:10.3390/microorganisms7120658)

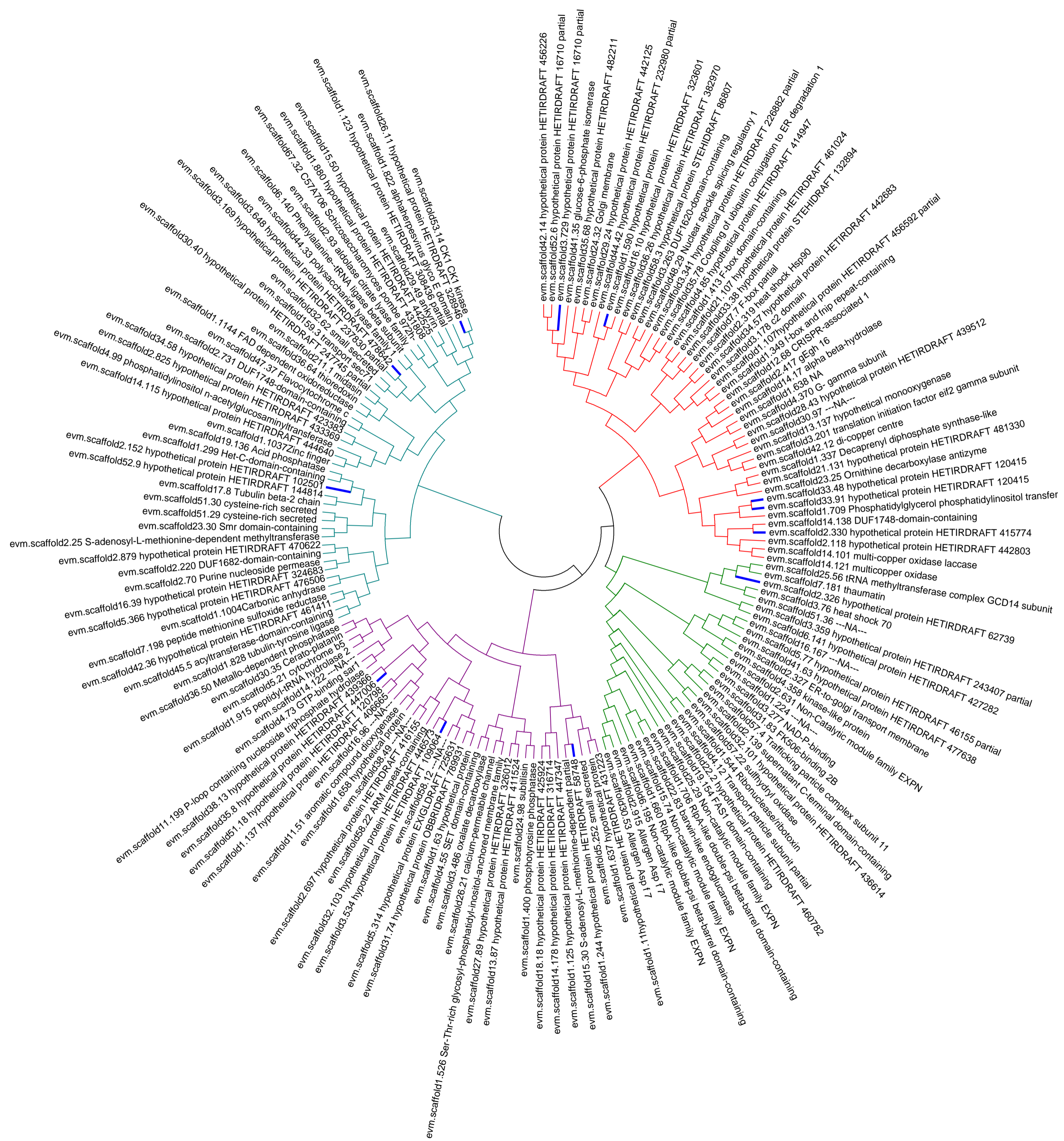

Supplement: Supplementary file 1 [file microorganisms-07-00658-s001.zip › Figure S1.pdf]
